# Assessment of differences in clinical consequences between rituximab and interferon recipients in multiple sclerosis patients after recovery from COVID-19

**DOI:** 10.1016/j.ibneur.2025.08.015

**Published:** 2025-10-17

**Authors:** Arash Musa AL-Rezai Aghdam, Shayan Sarkhosh, Zahra Farrokhi, Saleh Behzadi, Nazanin Moeini, Sahar Mehranfar

**Affiliations:** a Department of Neurology, Urmia University of Medical Sciences, Urmia, Iran; b Student Rsearch Committee, Urmia University of Medical Sciences, Urmia, Iran; c School of Medicine, Shahid Beheshti University of Medical Sciences, Tehran, Iran; d Student Research Committee, Rafsanjan University of Medical Sciences, Rafsanjan, Iran; e Student Research Committee, School of Medicine, Arak University of Medical Sciences, Arak, Iran; f Department of Genetics and Immunology, Faculty of Medicine, Urmia University of Medical Sciences, Urmia, Iran

**Keywords:** Multiple Sclerosis, COVID-19, Rituximab, Interferon, Disease-Modifying Therapies, Clinical Outcomes

## Abstract

**Background:**

Multiple sclerosis (MS) patients using disease-modifying treatments (DMTs) are at serious danger from the Coronavirus Disease 2019 (COVID-19) pandemic, which is brought on by the severe acute respiratory syndrome coronavirus-2 (SARS-CoV-2). This could make infections more severe. This study postulated that because of the immunosuppressive effects of Rituximab, MS patients treated with this medication would have worse clinical outcomes after recovering from COVID-19 than those treated with Interferon.

**Method:**

This cross-sectional study at Imam Khomeini Hospital, Urmia, Iran, included 34 MS patients with confirmed COVID-19 (2019–2022), treated with Interferon (n = 12) or Rituximab (n = 22). Demographic data, MS phenotype, Expanded Disability Status Scale (EDSS), COVID-19 severity, hospitalization duration, mortality, and reinfection rates were analyzed using SPSS version 27. Statistical comparisons used independent t-tests and Chi-square/Fisher’s exact tests (p < 0.05).

**Result:**

Patients had a mean age of 44.74 ± 8.85 years (range: 18–60). Rituximab-treated patients had significantly longer hospitalizations (6–10 days: 10 vs. 5 patients, 11–20 days: 5 vs. 0, p = 0.032, 95 % CI: 1.2–3.5), higher mortality (3 vs. 0, p = 0.041, odds ratio: 2.8, 95 % CI: 1.1–7.2), greater MS severity (EDSS≥2: 18 vs. 2, p = 0.028, 95 % CI: 1.3–4.1), and higher reinfection rates (8 vs. 0, p = 0.037, 95 % CI: 1.2–5.0) compared to Interferon-treated patients.

**Conclusion:**

Compared to interferon, rituximab was linked to worse clinical outcomes in MS patients after COVID-19 recovery, indicating that its use during pandemics should be used with caution.

## Introduction

Multiple sclerosis (MS) patients (Ge et al., 2020, Lu et al., 2020) and other immunocompromised individuals are at serious danger from the COVID-19 pandemic, which has been caused by the severe acute respiratory syndrome coronavirus-2 (SARS-CoV-2) since December 2019 (Wei et al., 2020, Ciotti et al., 2020, Castelo-Branco et al., 2020, Danza and Ruiz-Irastorza, 2013). MS, an autoimmune and neurological disease, is commonly treated with illness-modifying therapies (DMTs), such as Rituximab and Interferon, which change or block immune responses (Zabalza et al., 2021, Salter et al., 2021, Sepúlveda et al., 2021). Rituximab, a monoclonal anti-CD20 antibody, depletes B-cells, preventing antibody production and potentially impeding viral elimination (Chisari et al., 2022). Conversely, interferon beta stimulates the synthesis of antiviral genes, which prevents viral replication and enables immune priming (Freedman et al., 2021). These processes, especially in the context of COVID-19, raise concerns about the varying infection risks across MS patients.

Most people with MS are treated with disease-modifying therapies (DMTs), which are immunomodulators or immunosuppressive. They impact immune mechanisms such as immune cell subset depletion, alteration of immune cell function, inhibition of cell replication, and inhibition of immune cell trafficking. So, this treatment increases concerns about covid 19 in MS patients, enhancing the risk of infection and making the disease severe or fatal (Castelo-Branco et al., 2020, Danza and Ruiz-Irastorza, 2013, Winkelmann et al., 2016, Wijnands et al., 2017, Federation, 2020, Celius, 2017). Respiratory infections are more frequent in MS patients, with risks increasing with age, disability, and male sex (Winkelmann et al., 2016, Wijnands et al., 2017, Luna et al., 2020, Williamson and Berger, 2015).

Interferon is also crucial in modulation of the innate immune response and the amplification of antiviral signal transduction pathways that lead to reduced reinfection and mortality of MS patients who have recovered from COVID-19 (Mertowska et al., 2023). As a constituent of the body's first line of defense, interferon initiates several immune mechanisms that increase resistance against viral infections (Ortega-Prieto and Jimenez-Guardeño, 2024). Type I interferons, specifically interferon-beta, induce the expression of interferon-stimulated genes (ISGs), which inhibit viral replication by killing viral RNA, inhibiting protein synthesis, and preventing virion assembly (Mertowska et al., 2023). Furthermore, interferon puts immune cells such as macrophages and natural killer (NK) cells in a position to recognize and kill infected cells more quickly (Mihaescu et al., 2024).

In addition to its direct antiviral action, interferon enhances adaptive immune responses by facilitating antigen presentation and T lymphocyte activation (Gessani et al., 2014, Ye et al., 2019). The overall effects explain lower reinfection rates as a result of the immune system remaining vigilant against a second exposure to SARS-CoV-2 (Sun et al., 2021). Interferon's suppressive action on runaway inflammatory processes further prevents COVID-19 complications, which reduce the duration of hospitalization and mortality (Sun et al., 2021). With these protective mechanisms in view, interferon-treated MS patients would be expected to fare clinically better than rituximab-treated ones, which compromises immune surveillance by draining B-cells (Chattopadhyay et al., 2022). This distinction calls for a consideration of the immunomodulatory benefits of interferon in therapeutic intervention on MS patients in pandemic times (J. Gordon Betts et al., 2022).

DMTs, particularly B-cell-depleting treatments like Rituximab, can exacerbate infections, including COVID-19, and lead to an increased rate of hospitalization and mortality (Salter et al., 2021, Louapre et al., 2020, Simpson-Yap et al., 2021). Rituximab causes B cell depletion, which impairs antibody response and viral elimination, potentially aggravating COVID-19 in MS (Furlan et al., 2021). Interferon-beta, however, has the action of augmenting antiviral gene expression, inhibiting viral replication, and activating immune cells, resulting in better clinical outcomes after recovery from COVID-19 (Ortega-Prieto and Jimenez-Guardeño, 2024). These mechanisms explain the high rates of reinfection and mortality in Rituximab-treated COVID-19 patients and highlight the immunomodulatory benefit of Interferon during pandemic times. Despite all these differences, limited studies have compared clinical outcomes between MS patients following COVID-19 recovery under these treatments (Freedman et al., 2021, Moghadasi, 2020).

This study postulated that because of the immunosuppressive effects of Rituximab, MS patients treated with it would have worse clinical outcomes (hospitalization duration, death, MS severity, and reinfection rates) after recovering from COVID-19 than those treated with Interferon.

## Materials and methods

### Design

This cross-sectional analytical study was conducted at the neurology clinic of Imam Khomeini Hospital, Urmia, Iran, under the supervision of the MS Patient Support Association of West Azerbaijan Province. Patients diagnosed with MS (based on 2010 McDonald criteria or earlier standards) and confirmed COVID-19 (via PCR, 2019–2022) were included. Informed consent was obtained, and the study adhered to ethics code IR.UMSU.REC.1401.174.

### Study population

Inclusion criteria were: (Ge et al., 2020) confirmed MS diagnosis, (Lu et al., 2020) positive COVID-19 PCR test (2019–2022), and (Wei et al., 2020) treatment with Interferon or Rituximab. Exclusion criteria included non-compliance with these criteria or refusal to share medical data. Patients were divided into two groups: Rituximab-treated (n = 22) and Interferon-treated (n = 12). Data on vaccination status were not consistently available in hospital records.

### Data collection

Data on demographics, MS phenotype (Relapsing-Remitting MS [RRMS], Primary Progressive MS [PPMS], Secondary Progressive MS [SPMS], Progressive-Relapsing MS [PRMS]), baseline and post-recovery Expanded Disability Status Scale (EDSS)^2^ (Kurtzke, 1983, Meyer-Moock et al., 2014) (before Rituximab or Interferon administration), COVID-19 severity, hospitalization duration, mortality, and reinfection were extracted from hospital records and the MS Patient Support Association. PCR findings or patient reports were used to confirm reinfection. While all patients had confirmed COVID-19, variant classification (e.g., Alpha, Delta, Omicron) was not systematically recorded in medical records. Given the study period (2019–2022), patients were likely exposed to multiple SARS-CoV-2 variants. Phone interviews or in-person clinic visits were used to confirm the accuracy of the data.

### Outcomes

The length of hospital stay and the death rate were the main results. COVID-19 reinfection rates and MS severity (EDSS post-recovery) were secondary results.

### Statistical analysis

The descriptive data were presented with 95 % confidence intervals (mean ± SD, frequencies). Between-group comparisons used independent *t*-tests for continuous variables and Chi-square/Fisher’s exact tests for categorical variables (p < 0.05). Analyses were performed using SPSS version 27.

### Ethical issues

The study was approved by the Ethics Committee of Urmia University of Medical Sciences (IR.UMSU.REC.1401.174). Patient data were anonymized, and no additional costs or interventions were imposed.

## Result

### Baseline characteristics of the patients

Of 34 MS patients (mean age: 44.74 ± 8.85 years, range: 18–60), 71 % were female. The age range included younger patients with a mean age of 44.74 ± 8.85 years (range: 18–60). Baseline EDSS scores (pre-COVID-19) were comparable between groups (Rituximab: 1.8 ± 0.9, Interferon: 1.6 ± 0.7, p = 0.412). Among the 34 MS patients included in the study, 65 % (n = 22) were treated with Rituximab, while 35 % (n = 12) received Interferon.

### Hospitalization duration

**Fig. 1:**
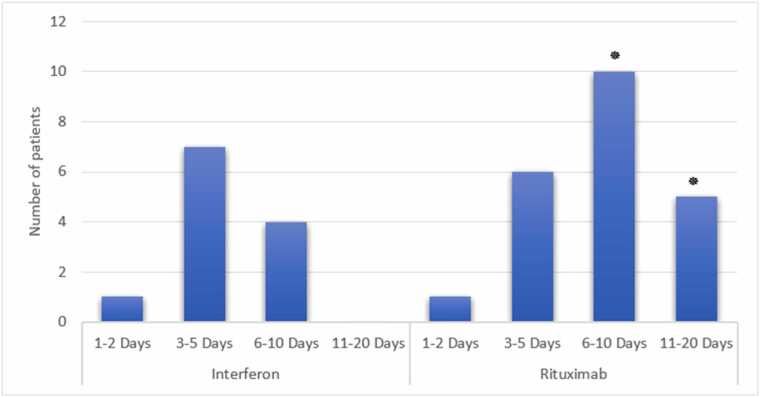
Duration of Hospitalization by Group (*: P < 0.05 compared to the corresponding group).

The length of hospitalization varied among the MS patients, with 41 % staying between 6–10 days, 15 % requiring 11–20 days of hospitalization, 38 % hospitalized for 3–5 days, and 6 % discharged within 1–2 days. Hospitalization durations were significantly longer in the Rituximab group (6–10 days: 10 patients, 11–20 days: 5 patients) compared to the Interferon group (6–10 days: 5 patients, 11–20 days: 0) (p = 0.032, Cohen’s d=0.82, 95 % CI: 1.2–3.5) (Fig. 1).

### Mortality rate

**Fig. 2:**
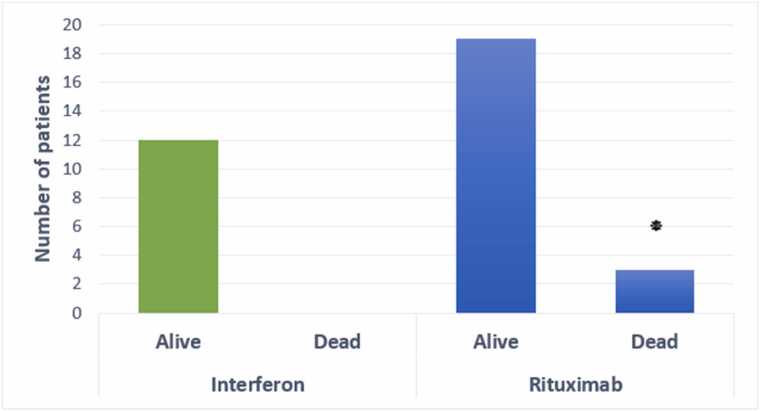
**Mortality Rate by Group.** Bar graph comparing mortality rates. *p < 0.05 (Fisher’s exact test).

Three Rituximab-treated patients died, compared to none in the Interferon group (p = 0.041, odds ratio: 2.8, 95 % CI: 1.1–7.2) (Fig. 2).

### MS severity (EDSS)

**Fig. 3:**
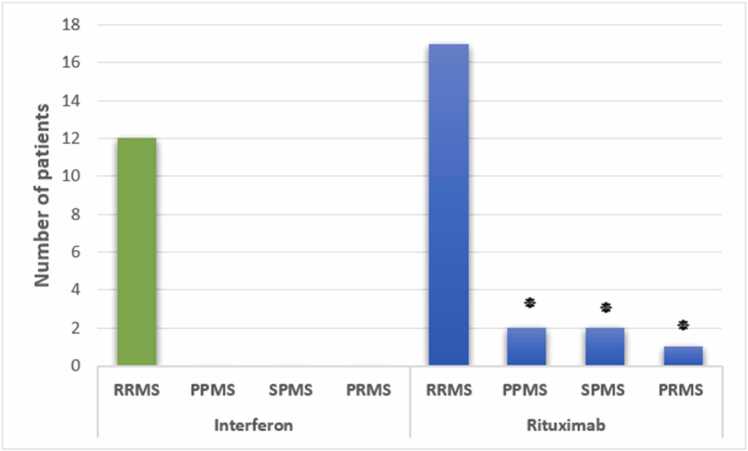
**MS Severity (EDSS) by Group** Bar graph comparing post-recovery EDSS scores among patients treated with Rituximab and Interferon. Significant differences in disability levels are indicated by *:*p < 0.05 (Chi-square test).*

The analysis of MS severity and disease phenotype among Rituximab- and Interferon-treated patients revealed significant differences in disability levels and disease progression. As presented in Fig. 3, all Interferon-treated patients had RRMS with mild disability, with 10 patients having an EDSS score of 1 and 2 patients having an EDSS score of 2. In contrast, Rituximab-treated patients exhibited a broader range of MS phenotypes, including PPMS (n = 2), SPMS (n = 2), and PRMS (n = 1), in addition to RRMS (n = 17). The EDSS indicated significantly greater disability in the Rituximab group, with 18 patients having EDSS scores ≥ 2 (p = 0.028, 95 % CI: 1.3–4.1).

**Fig. 4:**
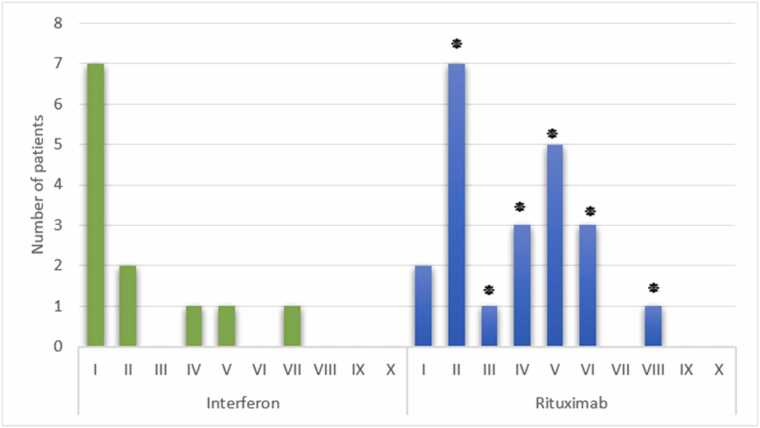
**Severity of MS Disease in the Study Groups**Comparison of MS disease severity across treatment groups. Higher EDSS scores and broader disease phenotypes observed in Rituximab-treated patients (*:p < 0.05 compared to the corresponding group).

Further examination of MS severity across study groups (Fig. 4) demonstrated that Rituximab-treated patients had higher EDSS scores across multiple severity levels (II–VIII, p < 0.05), indicating a greater burden of disability. In contrast, the Interferon group exhibited lower disability scores, with EDSS severity levels I vs. VII not showing significant differences (p = 0.214).

### Reinfection rates

**Fig. 5:**
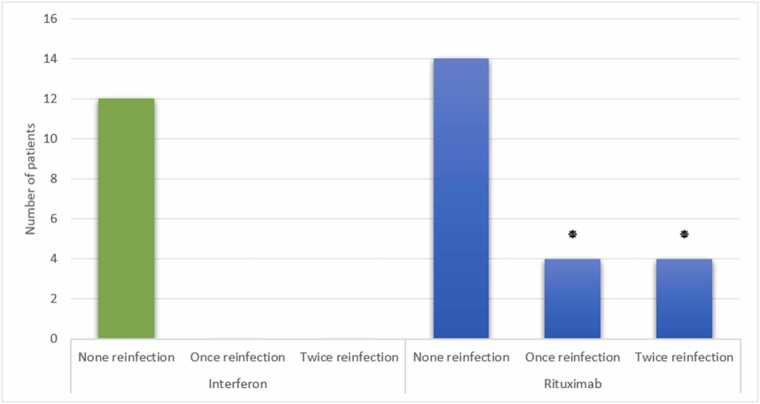
**Frequency of COVID Reinfection in the Study Groups** (*: P < 0.05 compared to the corresponding group; Fisher’s exact test).

Eight Rituximab-treated patients experienced reinfection (4 once, 4 twice), while none in the Interferon group did (p = 0.037, 95 % CI: 1.2–5.0) (Fig. 5).

## Discussion

**Fig. 6:**
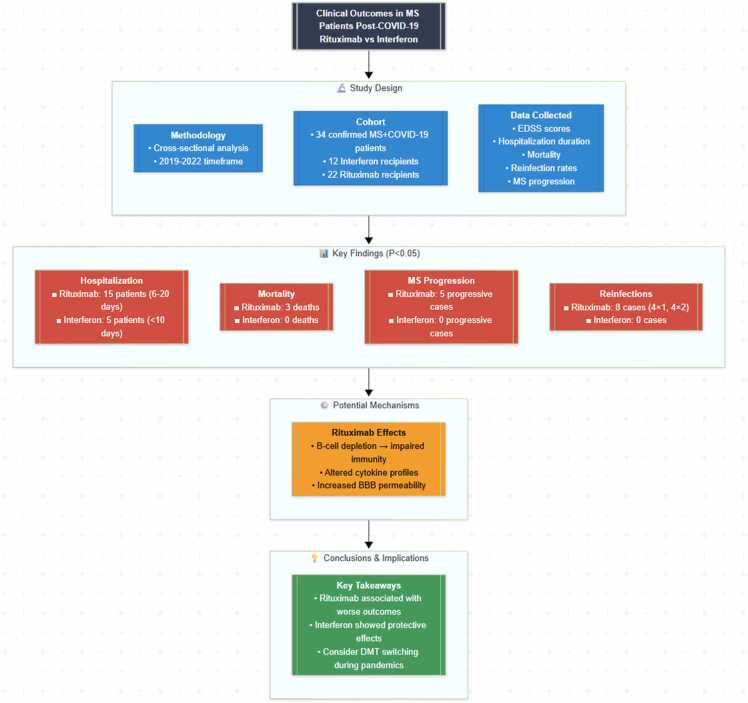
**Study Design and Outcomes (Mermaid Chart).** Caption: Flowchart illustrating the study design and key outcomes in MS patients post-COVID-19 recovery, comparing Rituximab (n = 22) and Interferon (n = 12) groups. Statistical significance indicated by *p < 0.05 (independent *t*-test or Chi-square/Fisher’s exact test).

Our data indicates that Rituximab, in contrast to Interferons, has been associated with greater rates of reinfection with COVID-19, increased risk of long-term hospitalization and mortality, and increased risk of MS aggravation. The flowchart in Fig. 6 provides a structured overview of the study methodology and key findings. These results are consistent with other research indicating that anti-CD20 medications like rituximab and ocrelizumab may make patients with rheumatic illnesses or multiple sclerosis more susceptible to and more severely affected by COVID-19. In addition, Rituximab may affect humoral and cellular immune responses to COVID-19 vaccination and therefore decrease protection against breakthrough infections (Schiavetti et al., 2022).

As seen in the findings, hospitalization times were longer in MS patients who were treated with Rituximab after contracting COVID-19 when compared to interferon treatment. In the Rituximab group, 10 patients spent six to ten days in the hospital, and five spent eleven to twenty days. In contrast, in the Interferon group, five spent six to ten days in the hospital, and none spent more than ten days. These results suggest that interferon therapy in MS patients infected with COVID-19 can reduce the time of hospitalization. This agrees with research by Freedman et al. (2021), which showed that interferon therapy reduced the hospital stay time below 12 days in a retrospective analysis of 630 mean aged 43 (Freedman et al., 2021). This observation was also recorded by Spelman et al. (2022) in light of the Swedish MS registry study, which found that Rituximab therapy had a 2.95-fold increased odds of hospitalization with COVID-19 compared to other disease-modifying drugs like Interferon beta and glatiramer acetate (Spelman et al., 2022).

Mortality analysis indicated no fatalities in interferon-treated MS patients, whereas three rituximab-treated patients died, showing considerably increased mortality (P < 0.05) in the rituximab group. Their finding is in line with Moghadasi et al. (2020), who showed that interferon treatment reduced COVID-19-associated mortality in MS patients (Moghadasi, 2020). During the COVID-19 pandemic, Rituximab-treated MS patients also had a very high mortality rate (P < 0.05), according to Iyer et al. (2021) (Iyer et al., 2021).

All of the patients receiving Interferon had RRMS, with the majority having a light severity (EDSS score I), according to a comparison of the type and severity of MS based on EDSS in individuals treated with Rituximab and Interferon after recovering from COVID-19. Contrarily, Rituximab-treated subjects presented with heterogenous forms of MS, including PPMS, SPMS, and PRMS, with most presenting with moderate to severe disability (EDSS score II or worse). The findings are consistent with the excellence of Interferon compared to rituximab in MS patients who contracted COVID-19 and are consistent with the Maarouf et al. (2020) research, where Rituximab was discovered to exacerbate MS severity during pandemic times (Maarouf et al., 2020). Similarly, in 2023, a study by Todorović et al. confirmed that Interferon reduced MS severity following recovery from COVID-19, consistent with our findings (Todorović et al., 2024).

All MS patients treated with interferon also did not experience COVID-19 reinfection, whereas in the Rituximab group, four patients received once and another four twice. All of these confirm the greater efficacy of interferon over Rituximab for the avoidance of COVID-19 reinfection in MS patients and concur with Barzegar et al. (2021) who conducted a systematic review of COVID-19 in MS patients (Barzegar et al., 2021).

Several mechanisms are responsible for the increased risk of poor outcomes among Rituximab-treated patients. Firstly, Rituximab depletes B cells that play a crucial role in antibody production against SARS-CoV-2, the agent causing COVID-19 (Chisari et al., 2022). B cells are also responsible for the antigen presentation to T cells, which are crucial in killing infected cells and generating long-term immunity. Therefore, Rituximab may impair adaptive and innate immunity and further complicate the body's ability to eliminate the virus and resist re-infection (Fong et al., 2023). Second, Rituximab has the potential to alter cytokine profiles and inflammatory response in MS patients (Kox et al., 2020), and induce an unbalanced immune response and increased tissue damage. This may escalate symptoms and complications of COVID-19 such as acute respiratory distress syndrome (ARDS), thrombosis, and multi-organ failure (Wang et al., 2021). Third, rituximab may have a direct effect on the central nervous system, increasing blood-brain barrier permeability and facilitating entry of SARS-CoV-2 into the brain, with potential induction of neuroinflammation and neurodegeneration, aggravating MS course and prognosis (Etemadifar et al., 2022).

Limitations include small sample size, retrospective design, lack of randomization/blinding, and potential confounders like age and comorbidities. Additionally, data on COVID-19 vaccination status were not consistently available in hospital records, limiting the ability to assess its impact on clinical outcomes. Given that Rituximab impairs vaccine response, unvaccinated patients may have experienced worse disease severity and higher reinfection rates. Moreover, this study did not incorporate long-term follow-up, preventing an evaluation of post-COVID MS progression and other potential long-term sequelae. Direct immune assessments, such as antibody titers and T-cell profiling, were also not performed, which limits mechanistic insights into Rituximab’s effects on viral clearance and immune response. Therefore, the results must be approached with caution and further research is needed to determine the causal relationship between Rituximab and COVID-19 outcomes in MS patients. The conclusions from the present study suggest that Rituximab is not the most optimal treatment for MS patients in the pandemic of COVID-19, especially in unvaccinated or high-risk exposed patients. Alternative treatments such as interferons have potentially better safety and efficacy profiles based on their antiviral and immunomodulatory effects and absence of interference with vaccines. However, starting, switching, or stopping rituximab must be individualized by balancing appropriately benefits and risks against patient wishes and values. MS patients under Rituximab treatment should be monitored closely for COVID-19 symptoms and complications and need to adhere to preventive measures such as mask-wearing, social distancing, and hand washing. They should be encouraged to receive COVID-19 vaccination and booster doses accordingly.

## Conclusion

Rituximab's prognosis in patients with MS following COVID-19 recovery is more dismal than Interferon. Physicians need to weigh these risks, particularly in unvaccinated patients, and balance against Interferon's antiviral protective advantage. Individualized clinical decision-making and adherence to preventive measures (e.g., vaccination, mask use) are the priority.

## CRediT authorship contribution statement

**Shayan Sarkhosh:** Writing – original draft, Investigation, Data curation. **Arash Musa AL-Rezai Aghdam:** Writing – review & editing, Project administration, Methodology. **Saleh Behzadi:** Writing – review & editing, Writing – original draft, Resources, Investigation. **Zahra Farrokhi:** Writing – original draft, Visualization. **Sahar Mehranfar:** Writing – review & editing, Formal analysis. **Nazanin Moeini:** Writing – review & editing.

## Ethics approval

Approved by Urmia University of Medical Sciences (IR.UMSU.REC.1401.174, January 2022).

## Funding

None.

## Declaration of Competing Interest

The authors declare that they have no known competing financial interests or personal relationships that could have appeared to influence the work reported in this paper.

## Footnotes

2 The Expanded Disability Status Scale (EDSS) is a standardized tool used to assess neurological disability in MS patients. MS phenotypes include Relapsing-Remitting MS (RRMS), characterized by alternating periods of disease activity and remission; Primary Progressive MS (PPMS), marked by continuous neurological decline without relapses; Secondary Progressive MS (SPMS), where initial relapsing-remitting disease transitions into progressive deterioration; and Progressive-Relapsing MS (PRMS), a rare form involving steady progression with superimposed relapses.
